# Factors Associated With Short-Term Complications After Percutaneous Endoscopic Gastrostomy Tube Insertion: A Retrospective Cohort Study

**DOI:** 10.7759/cureus.55741

**Published:** 2024-03-07

**Authors:** Mostafa Shehata, Ibrahim Al Hosani, Ishtiaq Ahmed, Heba Abu Alkas, Omar Khaddam, Abd Allah Aljanahi, Maryam Al Ahmad, Khalifa Al Tiniji, Yashbir Singh, Talha Malik

**Affiliations:** 1 Gastroenterology, Sheikh Shakhbout Medical City (SSMC), Abu Dhabi, ARE; 2 Internal Medicine, Sheikh Shakhbout Medical City (SSMC), Abu Dhabi, ARE; 3 Radiology, Mayo Clinic, Rochester, USA; 4 Gastroenterology, Mayo Clinic, Jacksonville, USA

**Keywords:** percutaneous endoscopic gastrostomy complication, percutaneous endoscopic gastrostomy, indication for gastrostomy, gastrostomy site infection, tube feeding, percutaneous endoscopic gastrostomy (peg) feeding

## Abstract

Background: Percutaneous endoscopic gastrostomy (PEG) tube placement is generally safe but is associated with a range of complications. Minor complications include infections, granuloma formation, leakage, and blockages, while major complications encompass aspiration pneumonia, hemorrhage, and more serious conditions such as necrotizing fasciitis and colonic fistula.

Aim: This study aimed to assess the rate of short-term complications within one month of endoscopic PEG insertion, focusing on their correlation with patient characteristics.

Methodology: This retrospective cohort study analyzed data from patients who underwent PEG insertion between January 2020 and December 2022. It evaluated the incidence of complications in relation to variables such as the indication for the procedure, the patient's immune status, albumin and CRP levels, and the setting of the procedure (inpatient vs. outpatient).

Results: The study included 121 patients, with a mean age of 69.73 years, comprising 71 males (58.7%) and 50 females (41.3%). Neurological indications accounted for 64.5% of the cases. Notably, 67.8% of the patients were immunocompromised. Within 30 days of PEG insertion, 16.5% experienced complications, including GI bleeding (4.1%), infection at the PEG site (11.6%), and peritonitis (0.8%). Complications were significantly higher in immunocompromised patients and those with non-neurological indications. Higher serum albumin and lower CRP levels were associated with fewer complications, though the association was not statistically significant.

Conclusion: The study highlights that gastrostomy site infection is the most common short-term complication following PEG insertion. Immune status and the reason for PEG insertion emerged as key factors influencing the likelihood of complications.

## Introduction

Since its introduction by Gauderer, the percutaneous endoscopic gastrostomy (PEG) tube has been a vital endoscopic intervention for patients requiring long-term enteral nutrition [1]. Inserted through the anterior abdominal wall into the stomach, the PEG tube provides an alternative to nasogastric feeding when the latter is infeasible or when assisted feeding is needed for more than 30 days [1,2].

Patients with PEG tubes tend to have lower complication rates and potentially a higher quality of life compared to those with nasogastric tubes, although no significant difference in mortality rates has been observed [3]. PEG tube placement is recommended when the need for enteral nutrition is either permanent or expected to exceed six weeks [4]. PEG tubes are applicable in a variety of conditions, including dysphagia due to cerebrovascular diseases, neurological disorders such as multiple sclerosis, and head and neck cancers [5], as well as in patients unable to maintain nutritional status orally, such as those with severe dementia.

Alternative methods of gastrostomy insertion, such as radiologically inserted tubes, exhibit comparable rates of major and minor complications to PEG tubes but have higher rates of tube dislodgement and replacement [6,7]. Despite the advantages of PEG feeding over long-term nasogastric feeding, such as improved patient comfort and a lower risk of aspiration, it is not without complications [8,9].

In terms of short-term complications, infection rates at the peristomal site vary widely, ranging from 2.3% to 27.9% [10]. Tube leakage, while less common, still occurs in up to 6.8% of cases [9,10]. Dislodgement happens in about 12.8% of cases, and minor bleeding episodes are reported to be between 1.4% and 9.7% [8-12]. Understanding the range of complications associated with PEG tubes is crucial, as it enables healthcare providers to offer comprehensive pre-insertion counseling to patients or their legal representatives, outlining both the risks and benefits. Furthermore, it aids in planning cost-effective support services and post-insertion management strategies. A better understanding of the predictors of complications can guide physicians in selecting suitable patients for PEG insertion and in taking measures to avoid such complications in those with one or more risk factors.

This study aims to identify risk factors associated with complications within the first 30 days post-PEG insertion, thereby establishing a protocol for clinical practice that could assist in making informed decisions about the procedure.

## Materials and methods

Study setting, design, and participants

A retrospective, single-center cohort study was conducted. The study included all patients over the age of 16 years who underwent PEG tube insertion at Sheikh Shakhbout Medical City from January 2020 to December 2022. All procedures were performed by a gastroenterologist under general anesthesia, either in the endoscopy unit or the operating theater. Patients received prophylactic antibiotics with cefazolin 1g IV, unless they had an allergy to cephalosporins, in which case amoxicillin-clavulanic acid was used as an alternative. Procedural consent was obtained from all participants or their legal guardians. The International Review Board of Sheikh Shakhbout Medical City reviewed and approved the study (approval number: SSMCREC-385)

PEG tube procedure

The PEG tube was inserted using the pull technique with a size 20 Fr tube. The procedure was performed by an endoscopist who is certified and licensed to practice by the Department of Health with the privilege of PEG insertion. A site with good transillumination was located in the stomach during gastroscopy for placement. The abdominal wall was marked, prepped in a sterile manner, and anesthetized with 2% lidocaine. The trocar needle was introduced through the abdominal wall into the stomach under direct endoscopic view. A snare was then introduced through the endoscope, opened in the gastric lumen, and closed around the guide wire. The endoscope and snare, along with the attached guide wire, were removed through the mouth. A skin incision was made at the needle insertion site, and the gastrostomy tube, lubricated and tied to the guide wire, was pulled through the mouth into the stomach. After removing the trocar needle, the gastrostomy tube was pulled out through the skin, the external bumper was attached, and the tube was cut to the appropriate length. The final position of the gastrostomy tube was confirmed by relook endoscopy, and the tension and compression of the abdominal wall by the PEG tube and external bumper were checked. The feeding tube was capped, and the site was cleaned and dressed. Feeding started after 6 hours post-procedure as clear fluid, then advanced to the feeding formula if tolerated.

Data

Short-term complications related to the PEG tube, defined as those occurring within 30 days post-insertion, included PEG site infection, peritonitis, and GI bleeding. Variables studied included demographics, indications for the procedure after a speech-language pathologist assessment (divided into neurological and non-neurological conditions), and patients' immune status, where diabetes, chronic kidney disease, and use of immunosuppressant medications will categorize the patients as immune suppressed. Albumin and CRP levels were collected within four weeks prior to the procedure. A PEG site infection was diagnosed based on clinical examination findings of erythema and discharge and confirmed by skin swab culture. Any case with overt GI bleeding that required upper GI endoscopy before discharge after gastrostomy tube insertion was counted. Data were also collected regarding the encounter with the PEG tube insertion and whether patients were referred from inpatient wards or the outpatient department. Medical records were retrospectively reviewed. Non-endoscopic PEG tube insertions and complications unrelated to the PEG tube insertion procedure were excluded from the study. The primary outcome was to assess the relationship between patient characteristics and short-term complications following PEG tube insertion. Logistic regression analysis was performed using univariate and multivariate models. Data were extracted from electronic health records. The data was de-identified and stored in a secure folder to ensure confidentiality.

Statistical methodology

Data were entered into the computer using SPSS Statistics version 22.0 (IBM Corp. Released 2013. IBM SPSS Statistics for Windows, Version 22.0. Armonk, NY: IBM Corp.). Data was entered as numerical or categorical, as appropriate. Two types of statistics were done:

Descriptive Statistics

Qualitative data were expressed as frequency and percent. Quantitative data was shown as mean, SD, median, range (min-max), and interquartile range.

Analytical Statistics

The Chi-square test was used to measure the association between qualitative variables. Fisher's exact test was used for 2x2 qualitative variables when more than 25% of the cells had an expected count of less than 5. Student's t-test was used to compare the mean and standard deviation of the test of quantitative normally distributed data, while the Mann-Whitney U test was used with data not normally distributed. Levene’s test for equality was used before the t-test to test the homogeneity of variance.

The logistic regression model was used to give an adjusted odds ratio and a 95% confidence interval for the effect of the different risk factors on the subject under study. A p-value will be considered statistically significant when it is less than 0.05.

## Results

A total of 121 patients were included in the study. Of these, 81 patients underwent PEG insertion while admitted for other reasons, such as aspiration pneumonia, post-stroke, or traumatic brain injury, and 40 patients received PEG insertions as referrals from the outpatient clinic. Ages ranged from 16 to 101 years, with a median age of 77 years. Fifty patients were female (41.3%), and 71 patients were male (58.7%). A neurological condition was the indication for PEG in 78 patients (64.5%), while a non-neurological condition was indicated in 43 patients (35.5%). There were 82 patients defined as immunocompromised (67.2%) and 39 as immunocompetent (32.8%) at the time of the procedure. CRP levels were measured in 113 patients, ranging from 1 to 112 mg/L, with a mean value of 21.9 mg/L. Serum albumin levels were measured in 91 patients, ranging from 14 to 39 g/L, with a mean of 26.5 g/L (Table 1).

**Table 1:** Characteristics data of PEG patients (n=121) PEG: percutaneous endoscopic gastrostomy, SD: standard deviation, IQR: interquartile range, CRP: C-reactive protein

		Value	Percent
Age (years)	Mean±SD	69.73±19.824	-
	Median (IQR)	77 (59.5-82.5)	-
	Min-max	16-101	-
Gender	Male	71	58.7
	Female	50	41.3
Indication for PEG	Neurological	78	64.5
	Non-neurological	43	35.5
Immune status	Immunocompetent	39	32.2
	Compromised	82	67.8
Overall complication	Yes	20	16.5
	No	101	83.5
Bleeding	Yes	5	4.1
	No	116	95.9
Infection at PEG	Yes	14	11.6
	No	107	88.4
Peritonitis	Yes	1	0.8
	No	120	99.2
Encounter of PEG	Yes	40	33.1
	No	81	66.9
CRP (n=113)	Mean±SD	21.9±22.3	-
	Median (IQR)	16 (6.5-27)	-
	Min-max	1-112	-
Albumin (n=91)	Mean±SD	26.58±5.996	-
	Median (IQR)	27 (22-31)	-
	Min-max	14-39	-
Total		121	100

Within 30 days post-procedure, overall complications occurred in 20 out of the 121 patients (16.5%), with five patients (4.1%) experiencing procedure-related GI bleeding, 14 patients (11.6%) developing PEG site infections, and one patient (0.8%) developing peritonitis. The only case in the studied cohort had peritonitis secondary to leakage, managed conservatively, and there was no statistical association with all the studied parameters.

Table 2 shows a comparison between patients who developed complications and those without, where the incidence of overall complications was significantly higher when PEG was indicated for non-neurological conditions (p=0.012) and when the patient was immunocompromised (p=0.004). CRP levels were numerically, but not statistically, higher in patients who experienced complications.

**Table 2:** Comparison of characteristics and variables of patients who had complications vs. not Immune status and non-neurological indications for PEG were more associated with 30 days post-procedure complications X2: Chi-square test, F: Fisher's exact test, T: Student's t-test, #: equal variances assumed, U: Mann-Whitney U test, *: significant, IQR: interquartile range (Q1-Q3), PEG: percutaneous endoscopic gastrostomy, CRP: C-reactive protein, SD: standard deviation

		Overall complication		Test of sig	p-value
		No	Yes		
Age (years)	N	101	20	U=1.47	0.142
	Mean±SD	68.28±20.515	77.05±14.133		
	Median (IQR)	75 (59.5-83.5)	80 (70-82.5)		
	Min-max	16-101	34-100		
Sex	Male	57 (80.3%)	14 (19.7%)	X^2^=1.26	0.26
	Female	44 (88%)	6 (12%)		
Indication	Neurological	70 (89.7%)	8 (10.3%)	X^2^= 6.259	0.012*
	Non-neurological	31 (72.1%)	12 (27.9%)		
Immune status	Immunocompetent	38 (97.4%)	1 (2.6%)	X^2^=8.134	0.004*
	Compromised	63 (76.8)	19 (23.2%)		
Encounter of PEG	Outpatient	32 (80%)	8 (20%)	X^2^=0.522	0.47
	Inpatient	69 (85.2%)	12 (14.8%)		
CRP	Mean±SD	19.96±18.654	31±33.789	U=1.1	0.27
	Median (IQR)	15 (6-25.5)	18.5 (9-38.25)		
	Min-max	1-93	5-112		
Albumin	Mean±SD	26.91±6.046	24.79±5.577	T=1.222#	0.225
	Median (IQR)	28 (22-32)	24 (20-29)		
	Min-max	14-39	17-35		

None of the studied parameters showed a significant association with post-procedure GI bleeding; however, it is worth mentioning that all five cases of GI bleeding occurred in patients for whom the PEG was inserted in an inpatient setting (Table 3).

**Table 3:** Comparison of patient characteristics of having post-procedure GI bleeding vs. not There was no association between the studied parameters and post-percutaneous endoscopic gastrotomy tube insertion GI bleeding F: Fisher's exact test, U: Mann-Whitney U test, *: significant, IQR: interquartile range (Q1-Q3), PEG: percutaneous endoscopic gastrostomy, CRP: C-reactive protein, SD: standard deviation, GI: gastrointestinal

		Bleeding		Test of sig	p-value
		No	Yes		
Age (years)	N	116	5	U=0.15	0.881
	Mean±SD	69.7±19.859	70.4±21.244		
	Median (IQR)	76.5 (59.25-82.75)	80 (52-84)		
	Min-max	16-101	34-87		
Sex	Male	67 (94.4%)	4 (5.6%)	F=0.978	0.3
	Female	49 (98%)	1 (2%)		
Indication	Neurological	75 (96.2%)	3 (3.8%)	F=0.45	0.83
	Non-neurological	41 (95.9%)	2 (4.7%)		
Immune status	Immunocompetent	38 (97.4%)	1 (2.6%)	F=0.357	0.913
	Compromised	78 (95.1%)	4 (4.9%)		
Encounter of PEG	Outpatient	40 (100%)	0 (0%)	F=2.575	0.169
	Inpatient	76 (93.8%)	5 (6.2%)		
CRP	N	108	5	U=0.237	0.812
	Mean±SD	21.79±22.12	23.8±29.198		
	Median (IQR)	16 (6.82-27.5)	15.1 (3.96-48)		
	Min-max	1-112	3-74		
Albumin	N	87	4	U=0.01	0.99
	Mean±SD	26.57±6.11	26.75±2.87		
	Median (IQR)	27 (22-32)	27 (23.75-29)		
	Min-max	14-39	23-29		

Analysis of PEG site infection showed that whenever PEG was inserted for a non-neurological condition and whenever the patient was immunocompromised, the incidence of 30-day PEG site infection increased significantly (p=0.003*, p=0.006*). CRP levels were numerically but not statistically higher, and serum albumin levels were numerically lower in patients who had a PEG site infection.

As shown in Table 4, based on unadjusted logistic regression analysis, undergoing PEG insertion for a non-neurological indication as opposed to a neurological one was associated with a more than threefold increase in the likelihood of a poor postoperative outcome (OR 3.39, 95% CI 1.28-9.44, p=0.016). However, after adjusting for variables with an effect size of more than 20%, the association, while still showing the same trend, was no longer statistically significant (OR 2.62, 95% CI 0.71-10.35, p=0.15). Similarly, undergoing PEG insertion during an immunocompromised state versus a period of normal immune status was associated with a substantially higher likelihood of poor postoperative outcomes, albeit with a very wide confidence interval (OR 11.46, 95% CI 2.24-210.03, p=0.02). Yet, this association also did not remain statistically significant after adjustment for variables, including all with effect sizes of more than 20%, and it continued to have a wide confidence interval (OR 4.75, 95% CI 0.77-93.29, p=0.1).

**Table 4:** Comparison of patient characteristics of having PEG site infection vs. not Indication for gastrostomy tube insertion and immune status was associated with gastrostomy site infection 30 days post-procedure X2: Chi-square test, F: Fisher's exact test, U: Mann-Whitney U test, *: significant, IQR: interquartile range (Q1-Q3), PEG: percutaneous endoscopic gastrostomy, CRP: C-reactive protein, SD: standard deviation

		Infection at PEG		Test of sig	p-value
		No	Yes		
Age (years)	N	107	14	U=1.48	0.138
	Mean±SD	68.49±20.386	79.21±11.396		
	Median (IQR)	76	79		
	Min-max	16-101	62-100		
Sex	Male	61 (85.9%)	10 (14.1%)	X^2^=1.06	0.303
	Female	46 (92%)	4 (8%)		
Indication	Neurological	74 (94.9%)	4 (51%)	X^2^=8.9	0.003*
	Non-neurological	33 (76.7%)	10 (23.3%)		
Immune status	Immunocompetent	39 (100%)	0 (0%)	X^2^=7.53	0.006*
	Compromised	68 (82.9%)	14 (17.1%)		
Encounter of PEG	Outpatient	32 (80%)	8 (20%)	X^2^=4.15	0.042*
	Inpatient	75 (92.6%)	6 (7.4%)		
CRP	N	99	14	U=0.85	0.395
	Mean±SD	20.48±20.216	31.79±33		
	Median (IQR)	16	15.5		
	Min-max	1-112	1-110		
Albumin	N	82	9	U=1.438	0.15
	Mean±SD	26.88±5.887	23.89±6.679		
	Median (IQR)	27.5	21		
	Min-max	14-39	17-35		

Finally, unadjusted logistic regression analysis suggested that higher CRP levels were associated with a marginally higher likelihood of poor postoperative outcomes in patients undergoing PEG insertion (OR 1.02, 95% CI 1.00-1.04, p=0.05). However, this association did not remain significant after adjusting for variables, including all with effect sizes of more than 20% (OR 1.01, 95% CI 0.99-1.04, p=0.29) (Table 5).

**Table 5:** Univariable and multivariable logistic regression analyses to identify predictors for complications post-PEG insertion Univariable and multivariable logistic regression analyses showed PEG insertion for a non-neurological indication, as opposed to a neurological one, was associated with a more than threefold increase in the likelihood of a poor postoperative outcome *Significant variables; p-value <0.05 squared variables were added to the model but were insignificant p-value >0.05

Dependent complication	X	Univariable		Multivariable	
		OR (95% CI)	p-value	OR (95% CI)	p-value
Gender	Female	-	-	-	-
	Male	1.80 (0.66-5.43)	0.265	1.49 (0.40-6.02)	0.555
Age	Years	1.03 (1.00-1.06)	0.077	1.01 (0.96-1.06)	0.819
Indication	Non-neurological	3.39 (1.28-9.44)	0.016*	2.62 (0.71-10.35)	0.151
	Neurological	-	-	-	-
Immune status	Compromised	11.46 (2.24-210.03)	0.020*	4.75 (0.77-93.29)	0.161
	Immunocompetent	-	-	-	-
CRP		1.02 (1.00-1.04)	0.055	1.01 (0.99-1.04)	0.286
Encounter of PEG	Outpatient	-	-	-	-
	Inpatient	0.70 (0.26-1.93)	0.472	0.84 (0.23-3.33)	0.790
Albumin		0.94 (0.85-1.04)	0.225	0.95 (0.85-1.06)	0.400

## Discussion

PEG is a widely used procedure for patients with intact GI function who require long-term enteral feeding. It is important to note that individuals needing PEG often suffer from chronic underlying health conditions and are generally in a fragile state. Although there are no universally accepted criteria for determining the necessity of PEG, the American Gastroenterological Association recommends its use in patients expected to survive beyond 30 days following the procedure [13-15]. Numerous studies have aimed to identify risk factors associated with PEG-related complications and mortality, with varied results [16-21]. Despite PEG generally being considered safer than radiological or surgical insertion methods, it has its own set of challenges. Complication rates vary from 13.2% to 42.9%, including bleeding, wound infections, tube blockages, leakage, aspiration pneumonia, perforation, and buried bumper syndrome [5]. Our study indicates that gastrostomy site infection is the most frequent short-term complication post-PEG. Immune status and the underlying reason for PEG insertion may act as predictive factors for these short-term complications, highlighting the necessity of careful patient evaluation and the consideration of potential risks before proceeding with PEG, thus emphasizing personalized medical decisions.

Extensive research has investigated risk factors for complications and mortality in patients undergoing PEG, identifying several potential predictors of adverse outcomes. These include elevated CRP levels, low serum albumin levels, hypernatremia, advanced age, malnutrition, comorbidities, and advanced stages of cancer [22-30]. However, discrepancies in study results can be attributed to factors such as short follow-up durations, which may not accurately reflect long-term outcomes, variations in sample sizes, and differences in the definitions of complications. Notably, low albumin and high CRP levels have been recognized as independent predictors of short-term mortality, a finding supported by the current study [31]. These markers indicate chronic inflammatory states that can negatively affect the immune system and metabolism, leading to decreased appetite and potential malnutrition. Patients with low albumin and high CRP levels are typically sicker and more vulnerable. Yet, in our study, CRP levels did not show a significant association with acute complications post-PEG, with most studies linking higher CRP levels to complications focusing on short- and long-term mortality, whereas our study only considered short-term, PEG-related complications [31].

Considering that PEG placement is not an emergency procedure, alternative, less invasive nutritional support options such as parenteral nutrition or a nasogastric catheter should be considered until the underlying acute condition has been thoroughly investigated and managed, prioritizing patient safety and optimized outcomes. The incidence of adverse events following PEG has been extensively studied, with Blomberg et al. reporting a one-month postoperative incidence of adverse events at 39%, which later decreased to 28% [32], illustrating the dynamic nature of complications in PEG patients and the influence of evolving conditions and care.

Neurological diseases are the most common indication for PEG placement, consistent with previous research [33-35], indicating that individuals with neurological conditions may be more prone to aspiration and therefore more likely to require PEG for enteral feeding. This necessitates early intervention to ensure adequate nutrition without the risk of aspiration. A notable finding in this study was the high prevalence of PEG site infections, at 21%, confirming the increased risk of infection, especially in the immediate post-procedural period [36,37]. The routine use of prophylactic antibiotics, such as ceftriaxone, during PEG insertion in our study center is a measure to mitigate this risk, following hospital protocols.

In our study, five patients (4.1%) experienced procedure-related GI bleeding that required upper GI endoscopy; however, none required endoscopic therapy or other modalities to control the bleeding. Lucendo et al.'s study, which included 6,233 patients, 3,665 of whom were undergoing antiplatelet treatment, suggested that PEG tube insertion is a safe procedure for patients on antiplatelet therapy, with no increased risk of procedural-related bleeding compared to control patients [38]. This finding is important as it may prevent exposing patients to potential thromboembolic risks due to medication withdrawal, which is often used for primary or secondary prevention of cardio and cerebrovascular diseases. However, more well-designed prospective research is needed to confirm these findings from predominantly retrospective data.

The limitations of this study must be acknowledged; the lack of a control group makes it challenging to estimate the odds ratio of adverse events in relation to potential risk factors accurately. The retrospective design introduces potential issues such as missing data from medical records and inaccuracies in assessing adverse events, which may affect the reported event rates. Additionally, the single-center nature of the study may limit its generalizability due to possible variations in population characteristics and healthcare practices across different settings. Therefore, while these findings provide valuable insights into PEG-related adverse events, caution should be exercised when generalizing them to broader populations and settings.

## Conclusions

PEG is considered a relatively safe procedure for long-term enteral feeding. The present study showed that major complications requiring interventions within 30 days of the procedure occur in less than 1% of patients. Our data also showed that gastrostomy site infection is the most common complication to occur in the short term after the procedure. The immune status of the patient and the indication for PEG insertion may predict short-term complications. Preoperatively defining and understanding these risk predictors would help improve care for those high-risk individuals and reduce their risk of developing post-percutaneous endoscopic gastrotomy complications.
